# Health-related quality of life and metabolic control in older people with diabetes mellitus^*^

**DOI:** 10.1590/1518-8345.8238.4936

**Published:** 2026-10-09

**Authors:** Ana Laura Galhardo Figueira, Carla Auxiliadora Margarido, Maria Angélica Andreotti Diniz, Marília Pilotto de Oliveira, Lucia Bacalhau, Luciana Kusumota

**Affiliations:** 1 Universidade de São Paulo, Escola de Enfermagem de Ribeirão Preto, PAHO/WHO Collaborating Centre for Nursing Research Development, Ribeirão Preto, SP, Brazil.; 2 Prefeitura Municipal de Lorena, Secretaria de Saúde, Lorena, SP, Brazil.; 3 Prefeitura Municipal de Sertãozinho, Serviço de Atendimento Móvel de Urgência, Sertãozinho, SP, Brazil.; 4 Universidade Católica Portuguesa, Faculdade de Ciências da Saúde e Enfermagem, Lisboa, Portugal.

**Keywords:** Quality of Life, Diabetes Mellitus, Aged, Primary Health Care, Public Health, Nursing Care

## Abstract

**Objective:**

to analyze health-related quality of life and its correlation with metabolic control in older people with diabetes mellitus treated in Primary Health Care.

**Method:**

observational, cross-sectional, and correlational study involving 264 older individuals with type 2 diabetes mellitus, treated in primary health care. Health-related quality of life was assessed using the Short Form Health Survey version 2 questionnaire, and metabolic control was assessed through laboratory tests (fasting blood glucose, HbA1c, lipid profile). Descriptive statistics and Spearman’s correlation (α=0.05) were applied.

**Results:**

participants had an average age of 70.23 years, 70.5% were women, and 86% had <9 years of education. The most compromised domain of health-related quality of life was pain, and the best performance was in emotional aspects. An inverse correlation was observed between general health status and HbA1c and between mental health and total cholesterol, triglycerides, and LDL.

**Conclusion:**

higher health-related quality of life scores, especially in the domains of general health and mental health, were associated with better metabolic control parameters, indicating the relevance of integrated approaches in the care of older people with diabetes mellitus in Primary Health Care.

## Highlights

**(1)** Aging and an increase in cases of type 2 diabetes mellitus.

**(2)** High prevalence of inadequate metabolic control.

**(3)** Quality of life is related to metabolic control.

**(4)** Implications for practice in Primary Health Care.

**(5)** Nursing is essential in promotion, prevention, and comprehensive care in Primary Health Care.

## Introduction

It is estimated that the world population over 60 years of age, which stood at 1 billion in 2020, will rise to 2.1 billion by 2050, with 80% of them living in developing countries^1^. This increase presents significant challenges for society and the healthcare system, especially in Brazil, where the population is aging at one of the fastest rates in the world^2^.

Older people are more vulnerable to developing chronic noncommunicable diseases (NCDs)^3^, such as diabetes mellitus (DM), whose prevalence increases with age. In its most recent publication, the International Diabetes Federation estimates that 589 million adults between the ages of 20 and 79 live with diabetes, equivalent to 1 in 9 people in this age group. The projection indicates that this number could reach 853 million by 2050^4^.

DM is a group of metabolic disorders characterized by underutilization and excessive production of glucose, leading to hyperglycemia^5^. Its treatment is based on a healthy diet, regular physical activity, maintaining adequate body weight, and abstaining from smoking. In addition, the use of oral medications and, in many cases, insulin is indicated for controlling blood glucose levels^6^.

When not properly controlled, DM can cause complications that negatively impact quality of life (QoL), manifesting as physical weakness, reduced functional capacity, pain, decreased vitality, social difficulties, and emotional instability^7^. In this context, QoL, understood as a broad and multidimensional construct, is analyzed in the field of health based on Health-Related Quality of Life (HRQoL), which focuses on the individual’s subjective perception of the effects of health status and care on their physical, emotional, and social functioning^8^. Thus, the present study adopts HRQoL as a theoretical framework to understand the impacts of DM and metabolic control on the daily lives of older people.

Care for elderly people with diabetes requires a comprehensive approach that considers medical, psychological, functional, and social aspects, which are fundamental for establishing realistic and individualized therapeutic goals, since these factors can interfere with self-management of the disease and influence HRQoL^9^. From this perspective, the Brazilian Diabetes Society establishes specific clinical and metabolic goals for this population, aiming to reduce complications, morbidity, and mortality^10^ ^-^ ^11^. However, a Brazilian study in Primary Health Care (PHC) indicates that a significant portion of older people with type 2 diabetes mellitus (T2DM) do not achieve the recommended glycemic goals, showing that metabolic control remains a challenge at this level of care^12^.

Given the complexity of the disease, caring for people with DM requires continuous, multidisciplinary monitoring with effective coordination of care. In this context, PHC stands out as the most appropriate level for longitudinal follow-up, with the Family Health Strategy (FHS) being the main policy for organizing PHC in Brazil, responsible for promotion, prevention, treatment, and rehabilitation actions^13^ ^-^ ^14^. Within this structure, the nursing team plays a central role in the continuity of care, especially in the development of educational actions, clinical monitoring, and the strengthening of self-care, contributing decisively to the comprehensive management of DM^15^.

Despite recognition of the relevance of HRQoL, there are still few studies that investigate, in an integrated manner, the relationship between different domains of HRQoL and multiple parameters of metabolic control in older adults with DM. This limits the systematic incorporation of this subjective dimension into care planning and qualifications.

Thus, given the relevance of HRQoL as a complementary dimension to clinical indicators and the evidence gap in PHC, the objective of this study was to analyze HRQoL and its correlation with metabolic control among older people with DM2 in PHC in a municipality in the interior of São Paulo, Brazil.

## Method

### Study design

Observational, analytical, and correlational study with a cross-sectional design. It is based on the Strengthening the Reporting of Observational Studies in Epidemiology (STROBE) guidelines.

### Location and period of data collection

The study was conducted in 11 FHS units in a municipality in the inland region of São Paulo, Brazil, between June and October 2022.

### Population

The population consisted of 1,113 older individuals who reported having DM in their individual records in the national health registry system, linked to one of the FHS units in the municipality in question. This number represents the total found in the electronic system in June 2021, the year in which the diagnostic survey of data for research planning was conducted.

### Selection criteria

People with a medical diagnosis of DM2, minimum age of 60 years, undergoing treatment with oral antidiabetic drugs (OAD) and/or insulin, preserved cognitive functions (assessed by the Mini-Mental State Examination - MMSE)^16^, and with verbal communication skills. The final MMSE score can range from 0 to 30 points^17^, and cut-off scores based on education level were used, considering the following points: 18 (illiterate), 21 (1-3 years of schooling), 24 (4-7 years of schooling), and 26 (> 7 years of schooling)^18^.

### Sample definition

Potential participants were identified through the national health registry system, using the following filters: age 60 years or older and DM. We chose to include all potentially eligible individuals during the collection period to maximize statistical power. Considering a population of 1,113 older people who reported having DM (total number before confirmation of the diagnosis of DM2 in the registry), a confidence level of 95% and a maximum tolerated error of 5% were established, resulting in an estimated minimum sample size of approximately 285 individuals. The final sample consisted of 264 participants, and it was not possible to increase this number due to the period previously defined in the research schedule and operational and logistical limitations. Although slightly lower than the estimated sample size, this sample remains sufficiently representative of the target population, enabling analyses with adequate statistical robustness. Furthermore, after reviewing the records of participants who agreed to participate in the research, it was found that not all had a confirmed medical diagnosis of DM2, which contributed to losses and reduced the number of eligible individuals among the 1,113 initially considered.

### Study variables

The study included the following variables: 1. Sociodemographic variables: gender, age, skin color, marital status, education level, current occupation, number of residents in the household, and monthly family income. 2. Health-Related Quality of Life (HRQoL): eight domains of the Short Form 36 Health Survey version 2 (SF-36v2^®^). 3. Metabolic control: fasting plasma glucose, glycated hemoglobin A_1c_ (HbA_1c_), total cholesterol, high-density lipoprotein (HDL) cholesterol, low-density lipoprotein (LDL) cholesterol, and triglycerides.

### Instruments used to collect information

Sociodemographic data were collected using an instrument designed for this purpose. HRQoL was assessed using the SF-36v2^®^, a reliable questionnaire sensitive to changes in health that assesses multiple domains of quality of life. Quick to administer, the questionnaire has been validated for the Brazilian population and has adequate psychometric properties, including internal consistency and construct validity, as demonstrated in studies with older adults^19^.

The SF-36 consists of 35 items and one question that asks about current health compared with that of a year ago. It assesses the perceived quality of life over the past four weeks, encompassing both negative (limitations) and positive (well-being) physical aspects and mental health. The items are distributed across eight domains: functional capacity (FC - 10 items), physical aspects (PA - 4), pain (2), general health status (GHS - 5), vitality (VT - 4), social aspects (SA - 2), emotional aspects (EA - 3), and mental health (MH - 5). These domains are organized into two components: physical (FC, PA, pain, and GHS) and mental (VT, SA, EA, and MH). In the present study, the average scores for each domain were reported, ranging from 0 (worst HRQoL) to 100 (best HRQoL)^20^. License number QM057367 was obtained for the use of SF-36v2^®^ from QualityMetric Inc.

Sociodemographic and HRQoL variables were self-reported by participants. Laboratory results for metabolic control assessment were obtained from electronic medical records, considering tests performed up to three months before or after data collection. There was no stratification by the exact date of the tests in this interval, which may introduce heterogeneity due to possible therapeutic changes, an aspect considered in the interpretation of the results. The tests were performed at a single reference laboratory within the municipal public health system, contributing to the standardization of analytical methods and reducing variability. Sample collection followed the service’s routine, was performed by trained professionals, and was conducted in accordance with institutional protocols, ensuring greater data uniformity.

To evaluate these latter variables, the standards established by consensus and/or guidelines were followed, with the following values being categorized as “normal” or “altered”: fasting plasma glucose (normal ≤ 130 mg/dL and altered > 130 mg/dL); HbA_1c_ (normal < 7.5% and altered ≥ 7.5%)^11^; total cholesterol (normal < 200 mg/dl and altered ≥ 200 mg/dl)^21^; triglycerides (normal < 150 mg/dl and abnormal ≥ 150 mg/dl)^22^; HDL (normal > 45 mg/dl and abnormal ≤ 45 mg/dl); LDL (normal < 100 mg/dl and abnormal ≥ 100 mg/dl)^21^.

### Data collection

Data collection was performed by a single researcher trained to reduce interobserver variability and information bias. The use of a validated instrument (SF-36v2®) and laboratory data recorded in the electronic system was intended to increase measurement reliability. The interviews were conducted at the participant’s home or, when possible, at the health unit, depending on availability and preference, in a private setting without third parties, present to minimize external interference and ensure privacy and comfort. The SF-36 is a structured self-reporting instrument that contributes to the standardization of responses regardless of the location of application.

The study instruments, printed on paper, were completed in ink and administered by the researcher in charge. Those who agreed to participate in the study were asked to sign an informed consent form (ICF), with a copy given to the participant.

### Data processing and analysis

The data were entered into Microsoft Excel^®^ spreadsheets with double data entry to ensure consistency. The analysis was conducted in R (version 4.3.1), using descriptive statistics to characterize sociodemographic variables, metabolic control parameters, and HRQoL domains, reported as absolute and relative frequencies, averages (with standard deviations), and medians (with minimum and maximum values). Bivariate analysis was performed using Spearman’s correlation coefficient, with a significance level set at 5% (α = 0.05). Variables with missing data were retained in the descriptive analysis with an indication of the valid number (n). No data imputation was performed; instead, case-by-case analysis was used to avoid introducing bias.

Multivariate models were not used due to variability in the number of valid cases across laboratory tests, the presence of missing data, and the cross-sectional nature of the study, which could compromise model stability and robustness. It is recognized that sociodemographic characteristics may act as potential confounding factors, an aspect considered in the interpretation of the results and discussed as a limitation of the study.

### Ethical considerations

The study was conducted in accordance with national and international ethical guidelines and approved by the Research Ethics Committee of the Ribeirão Preto School of Nursing, University of São Paulo, under Opinion No. 5,349,953.

## Results

During the data collection period, 1,113 participants eligible for the study were identified. The screening and inclusion flow is shown in Figure 1.

**Figure 1:**
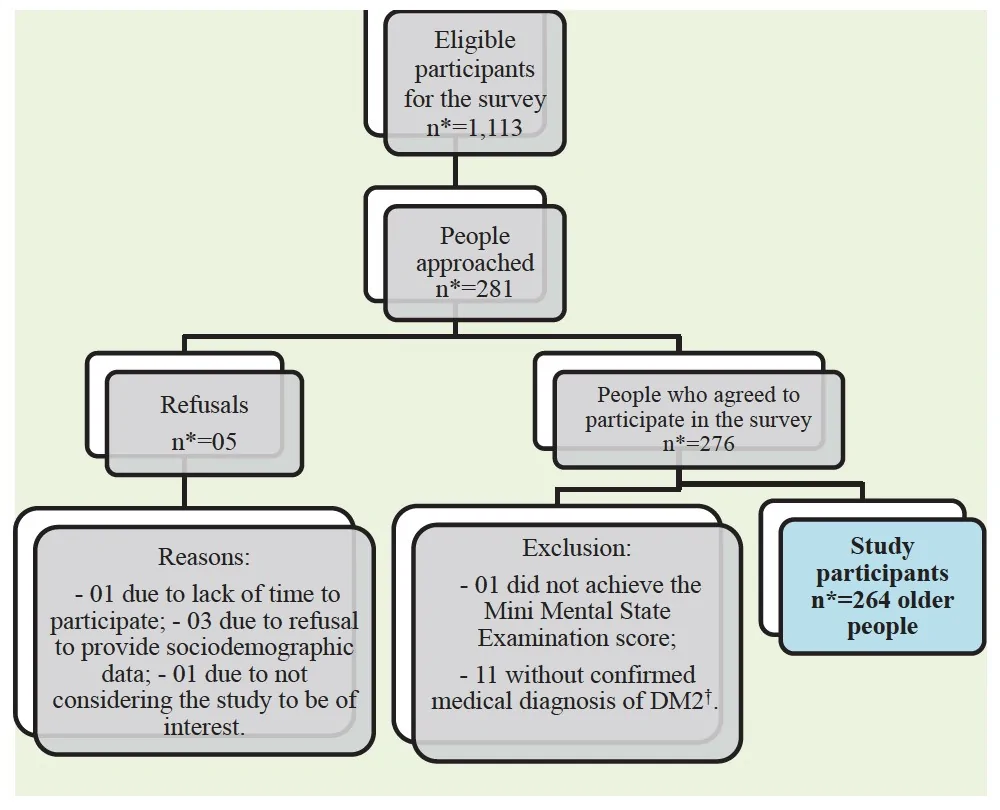
Process for eligibility and selection of study participants

*n = Number of respondents; ^†^DM2 = Type 2 diabetes mellitus

The sample was predominantly female, with an average age of 70.23 years (SD=6.84), most of whom were in the 60-69 age group. There was a prevalence of self-declared white individuals with low educational attainment who were retired or not engaged in paid work. Most lived with other residents, with an average of up to three people per household. Family income was mostly concentrated in the range of one to three minimum wages. Table 1 presents the sociodemographic characteristics of the participants.

In the HRQoL assessment, a variation in averages was observed across domains for older people with DM2, with greater impairment in the pain domain, and better HRQoL in the EA domain (Table 2).

**Table 1:** Sociodemographic characteristics of study participants (n = 264). Lorena, SP, Brazil, 2022

Sociodemographic variables	Average (SD*)	n^†^ (%^‡^)	Median (minimum-maximum)
**Gender** Male Female	-	78 (29.5) 186 (70.5)	-
**Age** (full years)	70.23 (6.84)	-	70.00 (60.00 - 94.00)
**Age range (in years)** 60-69 70-79 80-89 90-99	-	130 (49.2) 105 (39.8) 25 (9.5) 4 (1.5)	-
**Skin color** White Black Yellow Brown	-	171 (64.8) 36 (13.6) 1 (0.4) 56 (21.2)	-
**Marital status** Single Married/Cohabiting Separated/Divorced Widowed	-	28 (10.6) 126 (47.7) 31 (11.8) 79 (29.9)	-
**Education** (full years of study)	4.57 (3.52)	-	4.00 (0 - 19.00)
**Level of education** (in years) Low (< 9) Medium (9-12) High (>12)	-	227 (86.0) 28 (10.6) 9 (3.4)	-
**Current occupation** Retired but still employed Retired/pensioner/ work from home (unpaid) Unemployed	-	5 (1.9) 251 (95.1) 8 (3.0)	-
**Lives alone** Yes No	-	53 (20.1) 211 (79.9)	-
**Total number of people residing in the house** 1-3 people 4-6 people 7-9 people	2.55 (1.39)	214 (81.1) 45 (17.0) 5 (1.9)	2.00 (1.00- 8.00)
**Monthly family income** (n^†^=262) (BRL/BR$)^§^ < 1 salary 1-3 salaries 4 salaries or more	2.139.46 (1.566.81)	16 (6.1) 234 (89.3) 12 (4.6)	1.775,00 (0 - 13.000,00)

*SD = Standard deviation; ^†^n = Number of respondents; ^‡^% = Percentage; ^§^Current minimum wage = BRL/BR$ 1,212.00, Brazil, 2022

**Table 2:** Descriptive results of the eight domains of Health-Related Quality of Life of study participants (n = 264). Lorena, SP, Brazil, 2022

Domain	Average (SD*)	Median (minimum-maximum)
Functional Capacity	65.41 (28.37)	75.00 (0-100)
Physical Aspects	84.42 (30.84)	100.00 (0-100)
Pain	59.94 (31.20)	61.00 (0-100)
General Health Status	62.97 (24.06)	67.00 (0-100)
Vitality	64.54 (30.52)	75.00 (0-100)
Social Aspects	82.95 (25.74)	100.00 (0-100)
Emotional Aspects	88.57 (25.62)	100.00 (0-100)
Mental Health	71.50 (28.46)	80.00 (0-100)

*SD = Standard deviation

Analysis of the metabolic control results revealed changes in the averages values for most of the tests evaluated. It was observed that most participants had altered fasting blood glucose and LDL levels, indicating impaired control of these variables. Also noteworthy is the marked dispersion of fasting blood glucose values, as evidenced by the wide range between the minimum and maximum values (60-416 mg/dL), reflecting heterogeneity in glycemic control among the older individuals evaluated (Table 3).

**Table 3:** Characterization of metabolic control results for study participants (n = 264). Lorena, SP, Brazil, 2022

Metabolic control variables	Average (SD*)	n^†^ (%^‡^)	Median (minimum-maximum)
**Fasting Blood Sugar (mg/dl^§^) (n^†^=226)** Normal (<130) Altered (≥130)	151.71 (67.77)	108 (47.8) 118 (52.2)	132.00 (60.00-416.00)
**Glycated Hemoglobin (%^‡^) (n^†^=230)** Normal (<7.5) Altered (≥7.5)	7.96 (2.30)	124 (53.9) 106 (46.1)	7.20 (4.50-16.90)
**Total Cholesterol (mg/dl^§^) (n^†^=222)** Normal (<200) Altered(≥200)	199.11 (49.65)	115 (51.8) 107 (48.2)	196.50 (61.00-396.00)
**Triglycerides (mg/dl^§^) (n^†^=221)** Normal (<150) Altered (≥150)	169.20 (95.80)	114 (51.6) 107 (48.4)	147.00 (46.00-730.00)
**HDL (mg/d^§^) (n^†^=211)** Normal (>45) Altered(≤45)	50.15 (14.75)	125 (59.2) 86 (40.8)	49.00 (22.00-137.00)
**LDL (mg/dl^§^) (n^†^=212)** Normal (<100) Altered (≥100)	115.50 (43.12)	80 (37.7) 132 (62.3)	147.00 (46.00-305.00)

*SD = Standard deviation; ^†^n = Number of respondents; ^‡^% = Percentage, ^§^mg/dl = milligrams/deciliter

Statistical analysis revealed weak but statistically significant inverse correlations between specific domains of HRQoL and metabolic markers. Older people with DM2 who reported better perceptions of their GHS and MH had better laboratory test results, suggesting that higher HRQoL levels correlate with better metabolic control (Table 4).

**Table 4:** Correlation between Health-Related Quality of Life variables and metabolic control of study participants (n = 264). Lorena, SP, Brazil, 2022

Metabolic control variables	Functional Capacity	Physical aspects	Pain	General Health Status	Vitality	Social Aspects	Emotional Aspects	Mental Health
r^*^ (*p* ^*†*^ , - value)								
**Fasting Blood Sugar (n†=226)**	0.0891 (0.1819)	-0.0231 (0.6962)	-0.0178 (0.7907)	-0.1259 (0.0588)	-0.1006 (0.1317)	0.0008 (0.9903)	-0.0029 (0.9656)	-0.1266 (0.0573)
Glycated Hemoglobin (n†=230)	-0.0486 (0.4630)	-0.0994 (0.1328)	-0.1067 (0.1066)	-0.1525 (0.0207)	-0.1171 (0.0763)	-0.0861 (0.1932)	-0.0861 (0.1932)	-0.1044 (0.1145)
Total cholesterol (n†=222)	-0.0787 (0.2432)	-0.0629 (0.3509)	-0.0988 (0.1423)	-0.0545 (0.4190)	-0.0925 (0.1697)	-0.0976 (0.1474)	-0.0482 (0.4747)	-0.2635 (0.0100)
Triglycerides (n†=221)	-0.0346 (0.6091)	0.0258 (0.7026)	0.0369 (0.5856)	-0.0290 (0.6681)	-0.0229 (0.6681)	0.0323 (0.6333)	-0.1070 (0.1128)	-0.1711 (0.0108)
HDL (n†=211)	-0.0986 (0.1536)	-0.0986 (0.1536)	-0.0616 (0.3737)	-0.0139 (0.8408)	0.0044 (0.9489)	-0.0825 (0.2326)	-0.0196 (0.7776)	0.0013 (0.9849)
LDL (n†=212)	-0.0639 (0.3544)	-0.0515 (0.4561)	-0.0927 (0.1786)	-0.0201 (0.7709)	-0.0882 (0.2010)	-0.0545 (0.4302)	0.0407 (0.5557)	-0.2128 (0.0100)

^*^ r = Spearman’s correlation coefficient; ^†^p = Significance level (values of p ≤ 0.05 were considered statistically significant and are indicated with ^†^); ^‡^n = Number of respondents

## Discussion

This study resulted in three significant findings: (1) Older adults with DM2 had satisfactory HRQoL, although with moderate impairment in some domains, especially pain, while the best performance was observed in the AE domain; (2) Impaired metabolic control among participants; (3) Subjective aspects of health perception showed a statistical association with metabolic control parameters. However, due to the cross-sectional design, these findings do not allow causal inference, indicating only associations between HRQoL and metabolic control.

Regarding the first finding, the HRQoL domains generally presented high scores, with EA standing out as the best performer, while the pain domain was the most compromised, highlighting its negative impact on the HRQoL of older people with DM2 followed up in PHC. This result differs from international studies conducted with adults with DM2, in which the highest scores were concentrated in the MH domain and the lowest in PA, indicating a greater functional impact of the disease. Such differences may reflect age, sociocultural, clinical, and health service access differences, which influence perceptions of well-being and daily limitations^23^, particularly when comparing health systems with Brazilian PHC, which is guided by regular monitoring and care coordination.

Regarding the second finding, the results of this study corroborate the national and international literature by indicating that metabolic control in older people with DM2 tends to be unsatisfactory. A multicenter study conducted in China with 2,736 older individuals showed that only 23% achieved HbA1c < 7%, and 4.6% simultaneously achieved metabolic targets^24^. In Brazil, data from Ribeirão Preto (SP) revealed an average HbA1c of 7.2 ± 1.7%, with 74.1% of the older below 8%, indicating difficulties in metabolic management of this population^12^. The convergence of these findings suggests that inadequate metabolic control in elderly people with DM2 is a global challenge, but that, in Brazilian PHC, it may be related to the adoption of individualized clinical approaches, that are compatible with the needs and characteristics of the older person.

Regarding the third finding, which concerns the association between the physical aspects of HRQoL and metabolic control parameters, it was observed that, although aging and diabetes mellitus are associated with declines in physical function and a higher prevalence of chronic pain^25^ ^-^ ^27^, there were no significant correlations between the FC, PA, and pain domains and metabolic control. This finding may be related to regular follow-up in PHC, appropriate therapy, self-care strategies, or comorbidities, indicating that unfavorable physical conditions are not always associated with poorer metabolic control in this context.

Also in relation to the physical domains of HRQoL, EGS stands out, whose inverse correlation with HbA1c indicates that a more negative perception of health is associated with higher blood glucose levels, reflecting greater difficulty in maintaining metabolic control. This finding is corroborated by a study carried out in India with people with DM2, which, after six months of self-management education, showed a significant improvement in HbA1c levels and in the scores of the physical component of the SF-36, evidencing an association between glycemic control and perception of well-being, as well as by a study that identified worse physical quality of life among olderIranian people with poorly controlled diabetes^28^.

Regarding the mental aspects of HRQoL, the VT, SA, and EA domains did not correlate with metabolic control in older adults with DM2 in this study, although the literature points to an association between inadequate glycemic control and lower vitality scores, related to greater fatigue^29^. Evidence also indicates that the social participation of older people with DM is limited by factors such as mobility and self-care, which may indirectly influence metabolic control^30^, as well as depressive symptoms and diabetic stress, which compromise adherence to treatment^31^. An inverse correlation was observed between the MH domain and total cholesterol, triglyceride, and LDL levels, indicating an association between better MH scores and a more favorable lipid profile, but this does not allow causal inference given the cross-sectional design. Complementarily, a study with a different population identified that an unfavorable lipid profile was associated with a higher future risk of depression, anxiety, and stress-related disorders^32^.

The inverse relationship between GHS scores and HbA1c, as well as between the MH domain and total cholesterol, triglyceride, and LDL levels, suggests that subjective aspects of well-being are more closely associated with metabolic control than physical limitations. In this context, HRQoL plays a central role, as changes in health perception and emotional well-being can influence self-care^33^, including therapeutic adherence, resulting in better metabolic indicators^34^ ^-^ ^36^. Corroborating these findings, a meta-analysis of middle-aged and older adults with DM2 demonstrated a significant reduction in HbA1c following psychological interventions, indicating that strategies focused on psychological aspects contribute to glycemic control, although further studies are needed to identify the most effective approaches^37^.

In the context of PHC, the results highlight the importance of nursing as an integral part of a multidisciplinary team caring for older people with DM2, especially in longitudinal follow-up, clinical monitoring, and educational activities focused on self-care^38^. The identification of subjective aspects of HRQoL associated with metabolic control reinforces the strategic role of nursing in strengthening the bond and improving the quality of care provided.

However, metabolic control in PHC does not depend solely on the individual or subjective characteristics of older adults, but also on contextual factors related to service organization, such as the availability of laboratory tests, the regularity of clinical follow-up, continuity of care, and heterogeneity across units. These structural and organizational aspects can influence both metabolic indicators and perceptions of health and quality of life, reinforcing the need for comprehensive, contextualized approaches to the care of older adults with DM2 that articulate clinical, subjective, and organizational dimensions to promote better health outcomes.

One limitation of this study is the variability in methods and instruments used in the literature to assess health-related quality of life, which hinders comparability of results, despite the use of the SF-36, a validated and widely used instrument. However, because it is a generic instrument, its lower sensitivity to specific aspects of diabetes mellitus may have influenced the absence of correlations in some domains.

The analysis using available cases, without data imputation, may have introduced completeness bias, given the variation in the number of participants with laboratory test results. Besides, variability in the number of valid cases and the presence of missing data prevented the performance of multivariate analyses, limiting the ability to adjust for potential sociodemographic confounding factors. The absence of behavioral measures, such as adherence to treatment, diet, and physical activity, limits the interpretation of the findings, since these factors may mediate the relationship between HRQoL and metabolic control. Sample loss was also observed in relation to the initial calculation, which may have reduced statistical power, especially in correlational analyses with missing data.

Furthermore, the associations identified were weak in magnitude, although statistically significant, and should be interpreted with caution. The cross-sectional design also limits causal inferences. Despite these limitations, the study provided insight into local particularities, lending relevance to the findings in the context of PHC.

The findings of this study deepen our understanding of the relationship between HRQoL and metabolic control in older adults with DM2, contributing to the promotion of healthier aging and strengthening the evidence base, especially in the field of nursing, and potentially guiding more informed care practices in PHC. It is important to highlight the importance of person-centered approaches, the building of interpersonal bonds, which are essential for treatment adherence and better clinical outcomes^39^, and the importance of mental health and subjective perception of the disease in diabetes management, indicating the need for integrated therapeutic interventions that consider psychological and emotional factors in addition to clinical parameters.

The results reflect the reality of elderlyolder people with DM2 treated at PHC units in a municipality in the interior of São Paulo and may not be directly generalizable to contexts with different sociodemographic, organizational, and health access profiles. However, the similarity of the findings to those from national and international studies suggests that the observed relationships between HRQoL and metabolic control may also occur in other older populations with DM2.

## Conclusion

The results of the study indicated that older people with DM2 had satisfactory HRQoL, despite unsatisfactory metabolic control of important clinical parameters. Discrete but statistically significant associations were observed among specific domains of HRQoL, especially general health status and mental health, and metabolic markers. Although weak in magnitude, these associations indicate that more positive subjective perceptions of health and mental well-being were associated with better glycemic control and lipid profiles.

These findings reinforce the relevance of incorporating into the PHC assistential practice, a systematic approach to HRQoL and psychosocial aspects into primary care practice when monitoring older people with diabetes. Identifying negative perceptions of health and mental health impairment can serve as a clinical indicator of increased risk of metabolic imbalance, supporting earlier and more individualized interventions.

In the professional sphere, the strategic role of nursing and multidisciplinary teams in providing integrated care, combining clinical monitoring, educational activities, self-care support, and mental health care, is noteworthy. Such strategies can promote greater therapeutic adherence and improve outcomes of diabetes management in this population.

From a public policy perspective, the results point to the need to strengthen care models that value person-centered approaches, with integration between clinical care and psychosocial support, especially in the context of an aging population and the high prevalence of chronic diseases. Investments in training PHC teams and in interdisciplinary strategies can improve health outcomes and promote healthier aging among older adults with DM2.

## Data Availability

All data generated or analysed during this study are included in this published article.
