# Supplementary material for: Health-related quality of life and metabolic control in older people with diabetes mellitus
Source: Rev Lat Am Enfermagem. 2026 Oct 9;34:e4936. doi: 10.1590/1518-8345.8238.4936 (PMC13652534; doi:10.1590/1518-8345.8238.4936)
Supplement: Translation

# Translation: Qualidade de vida relacionada à saúde e controle metabólico de pessoas idosas com diabetes mellitus^*^

**DOI:** 10.1590/1518-8345.8238.4937

**Authors:** Ana Laura Galhardo Figueira, Carla Auxiliadora Margarido, Maria Angélica Andreotti Diniz, Marília Pilotto de Oliveira, Lucia Bacalhau, Luciana Kusumota

**Affiliations:** 1 Universidade de São Paulo, Escola de Enfermagem de Ribeirão Preto, Centro Colaborador da OPAS/OMS para o Desenvolvimento da Pesquisa em Enfermagem, Ribeirão Preto, SP, Brasil.; 2 Prefeitura Municipal de Lorena, Secretaria de Saúde, Lorena, SP, Brasil.; 3 Prefeitura Municipal de Sertãozinho, Serviço de Atendimento Móvel de Urgência, Sertãozinho, SP, Brasil.; 4 Universidade Católica Portuguesa, Faculdade de Ciências da Saúde e Enfermagem, Lisboa, Portugal.

**Keywords:** Qualidade de Vida, Diabetes Mellitus, Idoso, Atenção Primária à Saúde. Saúde Pública, Cuidados de Enfermagem.

## Abstract

**Método:**

estudo observacional, transversal e correlacional, com 264 pessoas idosas com diabetes mellitus tipo 2, atendidas na Atenção Primária à Saúde. A qualidade de vida relacionada à saúde foi avaliada por meio do questionário *Short Form Health Survey* versão 2, e o controle metabólico por meio de exames laboratoriais (glicemia de jejum, HbA1c e perfil lipídico). Foram aplicadas estatísticas descritivas e correlação de Spearman (α=0,05).

**Resultados:**

os participantes apresentaram média de idade de 70,23 anos, 70,5% eram mulheres e 86% tinham escolaridade <9 anos. O domínio mais comprometido da qualidade de vida relacionada à saúde foi dor, e o melhor desempenho foi em aspectos emocionais. Observou-se correlação inversa entre estado geral de saúde e HbA1c e entre saúde mental e colesterol total, triglicerídeos e LDL.

**Conclusão:**

maiores escores de qualidade de vida relacionada à saúde, especialmente nos domínios estado geral de saúde e saúde mental, mostraram correlação com melhores parâmetros de controle metabólico, indicando a relevância de abordagens integradas no cuidado às pessoas idosas com diabetes mellitus na Atenção Primária à Saúde.

## Destaques

**(1)** Envelhecimento e aumento de casos de diabetes mellitus tipo 2.

**(2)** Alta prevalência de controle metabólico inadequado.

**(3)** Qualidade de vida influencia o controle metabólico.

**(4)** Implicações para a prática na Atenção Primária à Saúde.

**(5)** Enfermagem é essencial na promoção, prevenção e cuidado integral na Atenção Primária à Saúde.

## Introdução 

Estima-se que a população mundial com mais de 60 anos, de 1 bilhão em 2020, passará para 2,1 bilhões até 2050, com 80% dela vivendo em países em desenvolvimento^1^. Esse aumento apresenta desafios significativos para a sociedade e para o sistema de saúde, especialmente no Brasil, onde o envelhecimento é um dos mais rápidos do mundo^2^.

A pessoa idosa é mais vulnerável ao desenvolvimento de Doenças Crônicas Não Transmissíveis (DCNTs)^3^, como o Diabetes Mellitus (DM), cuja prevalência aumenta com a idade. Em sua publicação mais recente, a *International Diabetes Federation* estima que 589 milhões de adultos entre 20 e 79 anos vivam com diabetes, o equivalente a 1 em cada 9 pessoas nessa faixa etária. A projeção indica que esse número poderá alcançar 853 milhões até 2050^4^.

O DM é um grupo de distúrbios metabólicos caracterizados pela subutilização e produção excessiva de glicose, levando à hiperglicemia^5^. Seu tratamento baseia-se em uma alimentação saudável, prática regular de atividade física, manutenção do peso corporal adequado e abstinência do tabagismo. Além disso, o uso de medicamentos orais e, em muitos casos, de insulina é indicado para o controle dos níveis de glicose no sangue^6^. 

Quando não adequadamente controlado, o DM pode ocasionar complicações que impactam negativamente a qualidade de vida (QV), manifestando-se por debilidade física, redução da capacidade funcional, dor, diminuição da vitalidade, dificuldades sociais e instabilidade emocional^7^. Nesse contexto, a QV, compreendida como um constructo amplo e multidimensional, é analisada no campo da saúde a partir da Qualidade de Vida Relacionada à Saúde (QVRS), que focaliza a percepção subjetiva do indivíduo sobre os efeitos da condição de saúde e do cuidado em seu funcionamento físico, emocional e social^8^. Assim, o presente estudo adota a QVRS como referencial teórico para compreender os impactos do DM e do controle metabólico no cotidiano das pessoas idosas. 

O cuidado à pessoa idosa com diabetes requer uma abordagem abrangente, que considere aspectos médicos, psicológicos, funcionais e sociais, fundamentais para o estabelecimento de metas terapêuticas realistas e individualizadas, uma vez que esses fatores podem interferir no autogerenciamento da doença e influenciar a QVRS^9^. Nessa perspectiva, a Sociedade Brasileira de Diabetes estabelece metas clínicas e metabólicas específicas para essa população, visando reduzir complicações, morbidade e mortalidade^10^ ^-^ ^11^. Contudo, estudo brasileiro na Atenção Primária à Saúde (APS) indica que uma parcela expressiva de pessoas idosas com Diabetes Mellitus tipo 2 (DM2) não alcança as metas glicêmicas recomendadas, evidenciando que o controle metabólico ainda representa um desafio nesse nível de atenção^12^.

Diante da complexidade da doença, o cuidado à pessoa com DM requer acompanhamento multiprofissional e contínuo, com coordenação eficaz da atenção. Nesse contexto, a APS destaca-se como o nível mais adequado para o seguimento longitudinal, sendo a Estratégia Saúde da Família (ESF) a principal política de organização da APS no Brasil, responsável por ações de promoção, prevenção, tratamento e reabilitação^13^ ^-^ ^14^. Inserida nessa estrutura, a equipe de enfermagem exerce papel central na continuidade do cuidado, especialmente no desenvolvimento de ações educativas, no monitoramento clínico e no fortalecimento do autocuidado, contribuindo de forma decisiva para o manejo integral do DM^15^.

Apesar do reconhecimento da relevância da QVRS, ainda são escassos os estudos que investigam, de forma integrada, a relação entre diferentes domínios da QVRS e múltiplos parâmetros do controle metabólico em pessoas idosas com DM. Isso limita a incorporação sistemática dessa dimensão subjetiva no planejamento e na qualificação do cuidado.

Assim, considerando a relevância da QVRS como dimensão complementar aos indicadores clínicos e à lacuna de evidências no contexto da APS, o objetivo deste estudo foi: analisar a QVRS e sua correlação com o controle metabólico de pessoas idosas com DM2 na APS de um município do interior de São Paulo, Brasil. 

## Método

### Delineamento do estudo

Estudo observacional, analítico e correlacional com delineamento transversal. Ele se baseia nas diretrizes do*Strengthening the Reporting of Observational Studies in Epidemiology* ****(STROBE).

### Local e período da coleta de dados

O estudo foi realizado em 11 unidades de ESF de um município do interior do estado de São Paulo, Brasil, entre junho e outubro de 2022.

### População

A população foi composta por 1.113 pessoas idosas que informaram possuir DM no cadastro individual no sistema nacional de registro de saúde, vinculados a uma das unidades de ESF do município em questão. Este número representa o total encontrado no sistema eletrônico no mês de junho de 2021, ano em que se realizou o levantamento diagnóstico dos dados para planejamento da pesquisa. 

### Critérios de seleção

Pessoas com diagnóstico médico de DM2, idade mínima de 60 anos, em tratamento medicamentoso com Antidiabético Oral (ADO) e/ou insulina, funções cognitivas preservadas (avaliadas pelo Miniexame do Estado Mental - MEEM)^16^, e com capacidade de comunicação verbal. O escore final do MEEM pode variar de 0 a 30 pontos^17^ e foram empregadas as notas de corte com base na escolaridade, sendo considerados os pontos: 18 (analfabetos), 21 (1-3 anos de escolaridade), 24 (4-7 anos de escolaridade) e 26 (> 7 anos de escolaridade)^18^.

### Definição da amostra

A identificação dos possíveis participantes foi realizada por meio do sistema nacional de registro de saúde, utilizando os filtros: idade igual ou superior a 60 anos e DM. Optou-se por incluir todos os possíveis indivíduos elegíveis no período de coleta, visando maximizar o poder estatístico. Considerando uma população de 1.113 pessoas idosas que informaram ter DM (número total antes da confirmação em registro do diagnóstico de DM2), estabeleceu-se nível de confiança de 95% e erro máximo tolerado de 5%, resultando em um tamanho amostral mínimo estimado de aproximadamente 285 indivíduos. A amostra final foi composta por 264 participantes, não sendo possível ampliar esse número em razão do período previamente definido no cronograma da pesquisa, bem como de limitações operacionais e logísticas. Apesar de ligeiramente inferior ao número estimado, esse tamanho amostral mantém representatividade suficiente para a população-alvo, permitindo a realização de análises com robustez estatística adequada. Ademais, após a verificação dos registros dos participantes que aceitaram integrar a pesquisa, constatou-se que nem todos apresentavam diagnóstico médico confirmado de DM2, o que contribuiu para as perdas e reduziu o número real de indivíduos elegíveis dentre os 1.113 inicialmente considerados.

### Variáveis do estudo

O estudo incluiu as seguintes variáveis: 1. Variáveis sociodemográficas: sexo, idade, cor da pele, estado civil, escolaridade, ocupação atual, número de moradores na residência, renda mensal familiar. 2. Qualidade de Vida Relacionada à Saúde (QVRS): oito domínios do *Short Form 36 Health Survey* versão 2 (SF-36v2^®^). 3. Controle metabólico: glicemia plasmática de jejum, hemoglobina glicada A_1c_ (HbA_1c_), colesterol total, colesterol de alta densidade (HDL), colesterol de baixa densidade (LDL) e triglicerídeos.

### Instrumentos utilizados para a coleta das informações

Os dados sociodemográficos foram coletados por meio de instrumento elaborado para este fim. A QVRS foi avaliada utilizando o SF-36v2^®^, questionário confiável e sensível a mudanças na saúde, que avalia múltiplos domínios da qualidade de vida. De aplicação rápida, o questionário possui validação para a população brasileira e apresenta propriedades psicométricas adequadas, como consistência interna e validade de constructo, também demonstradas em estudos com pessoas idosas^19^. 

O SF-36 é composto por 35 itens e uma questão que compara a saúde atual com a de um ano atrás. Avalia a qualidade de vida percebida nas últimas quatro semanas, englobando aspectos físicos e mentais, tanto negativos (limitações) quanto positivos (bem-estar). Os itens estão distribuídos em oito domínios: capacidade funcional (CF - 10 itens), aspectos físicos (AF - 4), dor (2), estado geral de saúde (EGS - 5), vitalidade (VT - 4), aspectos sociais (AS - 2), aspectos emocionais (AE - 3) e saúde mental (SM - 5). Esses domínios se organizam em dois componentes: físico (CF, AF, dor e EGS) e mental (VT, AS, AE e SM). No presente estudo, foram apresentados os escores médios de cada domínio, variando de 0 (pior QVRS) a 100 (melhor QVRS)^20^. A licença de número QM057367 foi obtida para o uso do SF-36v2^®^ a partir do QualityMetric Inc.

As variáveis sociodemográficas e de QVRS foram autorreferidas pelos participantes. Os resultados laboratoriais para avaliação do controle metabólico foram obtidos de prontuários eletrônicos, considerando exames realizados até três meses antes ou após a coleta de dados. Não houve estratificação pela data exata dos exames nesse intervalo, o que pode introduzir heterogeneidade decorrente de possíveis mudanças terapêuticas, aspecto considerado na interpretação dos resultados. Os exames foram realizados em um único laboratório de referência da rede pública municipal, contribuindo para a padronização dos métodos analíticos e redução da variabilidade. A coleta das amostras seguiu a rotina do serviço, realizada por profissionais treinados, conforme protocolos institucionais, assegurando maior uniformidade dos dados.

Para avaliar estas últimas variáveis, seguiram-se os padrões estabelecidos por consensos e/ou diretrizes, sendo categorizadas em “normal” ou “alterada”, os seguintes valores: glicemia plasmática de jejum (normal ≤ 130 mg/dl e alterada > 130 mg/dl); HbA_1c_ (normal < 7,5% e alterada ≥ 7,5%)^11^; colesterol total (normal < 200 mg/dl e alterada ≥ 200 mg/dl)^21^; triglicerídeos (normal < 150 mg/dl e alterada ≥ 150 mg/dl)^22^; HDL (normal > 45 mg/dl e alterada ≤ 45 mg/dl); LDL (normal < 100 mg/dl e alterada ≥ 100 mg/dl)^21^.

### Coleta de dados

A coleta de dados foi realizada por uma única pesquisadora treinada para reduzir variabilidade interobservador e viés de informação. O uso de um instrumento validado (SF-36v2®) e de dados laboratoriais registrados no sistema eletrônico buscou aumentar a confiabilidade das medidas. As entrevistas foram realizadas no domicílio do participante ou, quando possível, na unidade de saúde, conforme disponibilidade e preferência, em ambiente reservado e sem a presença de terceiros, a fim de minimizar interferências externas e garantir privacidade e conforto, sendo o SF-36 um instrumento estruturado de autorrelato que contribui para a padronização das respostas independentemente do local de aplicação. 

Os instrumentos do estudo, impressos em papel, tiveram as respostas preenchidas à tinta e foram aplicados pela pesquisadora responsável. Para os que concordaram em participar do estudo foi solicitada a assinatura do termo de consentimento livre e esclarecido (TCLE), ficando uma cópia com o participante. 

### Tratamento e análise dos dados

Os dados foram inseridos em planilhas do Microsoft Excel^®^ com dupla digitação para assegurar a consistência. A análise foi conduzida no *software* R (versão 4.3.1), utilizando estatística descritiva para caracterização das variáveis sociodemográficas, parâmetros de controle metabólico e domínios da QVRS, com apresentação em frequências absoluta e relativa, médias (com desvio-padrão) e medianas (com valores mínimo e máximo). A análise bivariada foi realizada por meio do Coeficiente de Correlação de Spearman, com nível de significância estabelecido em 5% (α = 0,05). Variáveis com dados ausentes foram mantidas na análise descritiva com indicação do número válido (n). Não foi realizada imputação de dados, optando-se por análise por caso disponível, para evitar introdução de viés.

Não foram empregados modelos multivariados em razão da variabilidade no número de casos válidos entre os diferentes exames laboratoriais, da presença de dados ausentes e da natureza transversal do estudo, fatores que poderiam comprometer a estabilidade e a robustez dos modelos. Reconhece-se que características sociodemográficas podem atuar como potenciais fatores de confusão, aspecto considerado na interpretação dos resultados e discutido como limitação do estudo.

### Considerações éticas 

O estudo foi conduzido de acordo com as diretrizes de ética nacionais e internacionais e aprovado pelo Comitê de Ética em Pesquisa da Escola de Enfermagem de Ribeirão Preto da Universidade de São Paulo, obtendo a aprovação sob o Parecer n.º 5.349.953. 

## Resultados

Durante o período da coleta de dados, foram elencados 1.113 participantes elegíveis para a pesquisa. O fluxo de rastreio e inclusão está apresentado na Figura 1.

**Figura 1:** Processo de elegibilidade e seleção dos participantes do estudo

*n = Número de respondentes; ^†^DM2 = Diabetes Mellitus tipo 2

A amostra foi predominantemente composta por mulheres, com idade média de 70,23 anos (DP=6,84), sendo a maioria pertencente à faixa etária de 60 a 69 anos. Observou-se prevalência de pessoas autodeclaradas brancas, com baixa escolaridade e aposentadas ou sem atividade remunerada. A maior parte residia com outros moradores, com média de até três pessoas por domicílio. A renda familiar concentrou-se, em sua maioria, na faixa de um a três salários-mínimos. A Tabela 1 apresenta a caracterização sociodemográfica dos participantes.

Na avaliação da QVRS, observou-se uma variação das médias nos domínios das pessoas idosas com DM2, com maior comprometimento do domínio dor, e melhor QVRS na avaliação do domínio AE (Tabela 2).

**Tabela 1:** Caracterização sociodemográfica dos participantes do estudo (n = 264). Lorena, SP, Brasil, 2022

Variáveis sociodemográficas	Média (DP*)	n^†^ (%^‡^)	Mediana (mínimo-máximo)

**Sexo**			
Masculino		78 (29,5)	
Feminino		186 (70,5)	

**Idade (anos completos)**	70,23 (6,84)		70,00 (60,00 – 94,00)

**Faixa etária (em anos)**			
60-69		130 (49,2)	
70-79		105 (39,8)	
80-89		25 (9,5)	
90-99		4 (1,5)	

**Cor da pele**			
Branca		171 (64,8)	
Preta		36 (13,6)	
Amarela		1 (0,4)	
Parda		56 (21,2)	

**Estado civil**			
Solteira(o)		28 (10,6)	
Casada(o)/Amasiada(o)		126 (47,7)	
Separada(o)/Divorciada(o)		31 (11,8)	
Viúva(o)		79 (29,9)	

**Escolaridade (anos completos de estudo)**	4,57 (3,52)		4,00 (0 – 19,00)

**Faixa de escolaridade (em anos)**			
Baixa (< 9)		227 (86,0)	
Média (9-12)		28 (10,6)	
Alta (>12)		9 (3,4)	

**Ocupação atual**			
Aposentada(o) mantendo vínculo empregatício		5 (1,9)	
Aposentada(o)/pensionista/trabalho em casa (sem remuneração)		251 (95,1)	
Desempregada(o)		8 (3,0)	

**Reside sozinho**			
Sim		53 (20,1)	
Não		211 (79,9)	

**Número total de pessoas residentes na casa**	2,55 (1,39)		2,00 (1,00-8,00)
1-3 pessoas		214 (81,1)	
4-6 pessoas		45 (17,0)	
7-9 pessoas		5 (1,9)	

**Renda mensal familiar (n¹=262) (em reais)§**	2.139,46 (1.566,81)		1.775,00 (0 – 13.000,00)
< 1 salário		16 (6,1)	
1-3 salários		234 (89,3)	
4 salários ou mais		12 (4,6)	


*DP = Desvio-padrão, ^†^n = Número de respondentes; ^‡^% = Percentagem, ^§^Salário-mínimo vigente = R$ 1.212,00, Brasil, 2022

**Tabela 2:** Resultados descritivos dos oito domínios da Qualidade de Vida Relacionada à Saúde dos participantes do estudo (n = 264). Lorena, SP, Brasil, 2022

Domínio	Média (DP*)	Mediana (mínimo-máximo)
Capacidade Funcional	65,41 (28,37)	75,00 (0-100)
Aspectos Físicos	84,42 (30,84)	100,00 (0-100)
Dor	59,94 (31,20)	61,00 (0-100)
Estado Geral de Saúde	62,97 (24,06)	67,00 (0-100)
Vitalidade	64,54 (30,52)	75,00 (0-100)
Aspectos Sociais	82,95 (25,74)	100,00 (0-100)
Aspectos Emocionais	88,57 (25,62)	100,00 (0-100)
Saúde Mental	71,50 (28,46)	80,00 (0-100)

^*^ DP = Desvio-padrão

A análise dos resultados do controle metabólico revelou alterações nas médias da maioria dos exames avaliados. Observou-se que grande parte dos participantes apresentava níveis alterados de glicemia de jejum e LDL, indicando comprometimento no controle dessas variáveis. Destaca-se ainda a acentuada dispersão dos valores de glicemia de jejum, evidenciada pela ampla variação entre os valores mínimo e máximo (60-416 mg/dl), o que reflete heterogeneidade no controle glicêmico entre as pessoas idosas avaliadas (Tabela 3).

**Tabela 3:** Caracterização dos resultados do controle metabólico dos participantes do estudo (n = 264). Lorena, SP, Brasil, 2022

Variáveis de controle metabólico	Média (DP*)	n^†^ (%^‡^)	Mediana (mínimo-máximo)

**Glicemia de Jejum (mg/dl^§^) (n^†^=226)**	151,71 (67,77)		132,00 (60,00-416,00)
Normal (<130)		108 (47,8)	
Alterado (≥130)		118 (52,2)	

**Hemoglobina Glicada (%^‡^) (n^†^=230)**	7,96 (2,30)		7,20 (4,50-16,90)
Normal (<7,5)		124 (53,9)	
Alterado (≥7,5)		106 (46,1)	

**Colesterol Total (mg/dl^§^) (n^†^=222)**	199,11 (49,65)		196,50 (61,00-396,00)
Normal (<200)		115 (51,8)	
Alterado (≥200)		107 (48,2)	

**Triglicerídeos (mg/dl^§^) (n^†^=221)**	169,20 (95,80)		147,00 (46,00-730,00)
Normal (<150)		114 (51,6)	
Alterado (≥150)		107 (48,4)	

**HDL (mg/d^§|^) (n^†^=211)**	50,15 (14,75)		49,00 (22,00-137,00)
Normal (>45)		125 (59,2)	
Alterado (≤45)		86 (40,8)	

**LDL (mg/dl^§^) (n^†^=212)**	115,50 (43,12)		147,00 (46,00-305,00)
Normal (<100)		80 (37,7)	
Alterado (≥100)		132 (62,3)	

*DP = Desvio-padrão; ^†^n = Número de respondentes; ^‡^% = Percentagem; ^§^mg/dl = miligramas/decilitro

A análise estatística revelou correlações inversas de magnitude fraca, embora estatisticamente significativas, entre domínios específicos da QVRS e marcadores metabólicos. Pessoas idosas com DM2 com melhores percepções sobre seu EGS e SM apresentaram melhores resultados nos exames laboratoriais, sugerindo que níveis mais elevados de QVRS se correlacionam a um controle metabólico mais adequado (Tabela 4).

**Tabela 4:** Correlação entre as variáveis Qualidade de Vida Relacionada à Saúde e controle metabólico dos participantes do estudo (n = 264). Lorena, SP, Brasil, 2022

Variáveis de controle metabólico	Capacidade Funcional	Aspectos Físicos	Dor	Estado Geral de Saúde	Vitalidade	Aspectos Sociais	Aspectos Emocionais	Saúde Mental
r^*^ (*p* ^*†*^ , - valor)								
Glicemia de Jejum (n^‡^=226)	0,0891 (0,1819)	-0,0231 (0,6962)	-0,0178 (0,7907)	-0,1259 (0,0588)	-0,1006 (0,1317)	0,0008 (0,9903)	-0,0029 (0,9656)	-0,1266 (0,0573)
Hemoglobina Glicada (n^‡^=230)	-0,0486 (0,4630)	-0,0994 (0.1328)	-0,1067 (0,1066)	-0,1525 (0,0207†)	-0,1171 (0,0763)	-0,0861 (0,1932)	-0,0861 (0,1932)	-0,1044 (0,1145)
Colesterol total (n^‡^=222)	-0,0787 (0,2432)	-0,0629 (0,3509)	-0,0988 (0,1423)	-0,0545 (0,4190)	-0,0925 (0,1697)	-0,0976 (0,1474)	-0,0482 (0,4747)	-0,2635 (0,0100†)
Triglicerídeos (n^‡^=221)	-0,0346 (0,6091)	0,0258 (0,7026)	0,0369 (0,5856)	-0,0290 (0,6681)	-0,0229 (0,6681)	0,0323 (0,6333)	-0,1070 (0,1128)	-0,1711 (0,0108†)
HDL (n^‡^=211)	-0,0986 (0,1536)	-0,0986 (0,1536)	-0,0616 (0,3737)	-0,0139 (0,8408)	0,0044 (0,9489)	-0,0825 (0,2326)	-0,0196 (0,7776)	0,0013 (0,9849)
LDL (n^‡^=212)	-0,0639 (0,3544)	-0,0515 (0,4561)	-0,0927 (0,1786)	-0,0201 (0,7709)	-0,0882 (0,2010)	-0,0545 (0,4302)	0,0407 (0,5557)	-0,2128 (0,0100†)

^*^ r = Coeficiente de correlação de Spearman; ^†^p = Nível de significância (valores de p ≤ 0,05 foram considerados estatisticamente significativos e estão indicados com ^†^); ^‡^n = Número de respondentes

## Discussão

Este estudo resultou em três achados consideráveis: (1) As pessoas idosas com DM2 apresentaram uma QVRS satisfatória, embora com comprometimento moderado em alguns domínios, especialmente dor, enquanto o melhor desempenho foi observado no domínio AE; (2) Controle metabólico comprometido entre os participantes; (3) Os aspectos subjetivos da percepção de saúde mostraram associação estatística com parâmetros do controle metabólico. Contudo, em razão do delineamento transversal, esses achados não permitem estabelecer relações causais, indicando apenas a existência de associações entre a QVRS e o controle metabólico.

Em relação ao primeiro achado, os domínios da QVRS apresentaram, em geral, escores elevados, com destaque para AE como o de melhor desempenho, enquanto o domínio dor foi o mais comprometido, evidenciando seu impacto negativo na QVRS de pessoas idosas com DM2 acompanhadas na APS. Esse resultado difere de estudos internacionais conduzidos com adultos com DM2, nos quais os maiores escores concentraram-se no domínio SM e os menores em AF, indicando maior impacto funcional da doença. Tais divergências podem refletir diferenças etárias, socioculturais, clínicas e de acesso aos serviços de saúde, que influenciam a percepção de bem-estar e as limitações cotidianas^23^, particularmente quando se compara sistemas de saúde com a APS brasileira, orientada pelo acompanhamento regular e pela coordenação do cuidado.

No que se refere ao segundo achado, os resultados do presente estudo corroboram a literatura nacional e internacional ao indicar que o controle metabólico em pessoas idosas com DM2 tende a ser insatisfatório. Estudo multicêntrico realizado na China, com 2.736 pessoas idosas, mostrou que apenas 23% atingiram HbA1c < 7% e 4,6% alcançaram simultaneamente as metas metabólicas^24^. No Brasil, dados de Ribeirão Preto (SP) revelaram média de HbA1c de 7,2 ± 1,7%, com 74,1% dos idosos abaixo de 8%, evidenciando as dificuldades no manejo metabólico dessa população^12^. A convergência desses achados sugere que o controle metabólico inadequado das pessoas idosas com DM2 é um desafio global, mas que, na APS brasileira, pode estar relacionado à adoção de condutas clínicas individualizadas, compatíveis com as necessidades e características da pessoa idosa.

Em relação ao terceiro achado, que diz respeito à associação entre os aspectos físicos da QVRS e os parâmetros de controle metabólico, observou-se que, embora o envelhecimento e o diabetes mellitus estejam associados a declínios na função física e a maior prevalência de dor crônica^25^ ^-^ ^27^, não houve correlações significativas entre os domínios CF, AF e dor e o controle metabólico. Tal achado pode estar relacionado ao acompanhamento regular na APS, ao uso de terapêutica adequada, a estratégias de autocuidado ou à influência de comorbidades, indicando que condições físicas desfavoráveis nem sempre se refletem em pior controle metabólico nesse contexto.

Ainda em relação aos domínios físicos da QVRS, destaca-se o EGS, cuja correlação inversa com a HbA1c indica que uma percepção mais negativa da saúde está associada a níveis mais elevados de glicose no sangue, refletindo maior dificuldade em manter o controle metabólico. Esse achado é corroborado por estudo realizado na Índia com pessoas com DM2, que, após seis meses de educação em autogerenciamento, apresentou melhora significativa nos níveis de HbA1c e nos escores do componente físico do SF-36, evidenciando associação entre controle glicêmico e percepção de bem-estar, bem como por pesquisa que identificou pior qualidade de vida física entre pessoas idosas iranianas com diabetes mal controlado^28^.

Quanto aos aspectos mentais da QVRS, os domínios VT, AS e AE não apresentaram correlação com o controle metabólico das pessoas idosas com DM2 neste estudo, embora a literatura aponte associação entre controle glicêmico inadequado e piores escores de vitalidade, relacionados à maior fadiga^29^. Evidências indicam ainda que a participação social de pessoas idosas com DM é limitada por fatores como mobilidade e autocuidado, podendo influenciar indiretamente o controle metabólico^30^, assim como sintomas depressivos e estresse diabético, que comprometem a adesão ao tratamento^31^. Observou-se correlação inversa entre o domínio SM e os níveis de colesterol total, triglicerídeos e LDL, indicando associação entre melhores escores de SM e perfil lipídico mais favorável, sem permitir inferência causal, considerando o delineamento transversal. De forma complementar, estudo com população distinta identificou que um perfil lipídico desfavorável associou-se a maior risco futuro de depressão, ansiedade e transtornos relacionados ao estresse^32^.

A relação inversa entre os escores de EGS e HbA1c, bem como entre o domínio SM e os níveis de colesterol total, triglicerídeos e LDL, sugere que aspectos subjetivos do bem-estar estão mais associados ao controle metabólico do que às limitações físicas. Nesse contexto, a QVRS assume papel central, pois alterações na percepção de saúde e no bem-estar emocional podem influenciar o autocuidado^33^, incluindo a adesão terapêutica, refletindo-se em melhores indicadores metabólicos^34^ ^-^ ^36^. Corroborando esses achados, uma meta-análise com adultos de meia-idade e idosos com DM2 demonstrou redução significativa da HbA1c após intervenções psicológicas, indicando que estratégias voltadas aos aspectos psicológicos contribuem para o controle glicêmico, embora ainda sejam necessários estudos para identificar as abordagens mais eficazes^37^.

No âmbito da APS, os resultados ressaltam a importância da atuação da enfermagem, como parte integrante de uma equipe multidisciplinar no cuidado à pessoa idosa com DM2, especialmente no acompanhamento longitudinal, no monitoramento clínico e nas ações educativas voltadas ao autocuidado^38^. A identificação de aspectos subjetivos da QVRS associados ao controle metabólico reforça o papel estratégico da enfermagem no fortalecimento do vínculo e na qualificação do cuidado ofertado.

Contudo, o controle metabólico na APS não depende somente das características individuais ou subjetivas das pessoas idosas, mas também de fatores contextuais relacionados à organização dos serviços, como a disponibilidade de exames laboratoriais, a regularidade do seguimento clínico, a continuidade do cuidado e a heterogeneidade entre as unidades. Esses aspectos estruturais e organizacionais podem influenciar tanto os indicadores metabólicos quanto a percepção de saúde e qualidade de vida, reforçando a necessidade de abordagens integrais e contextualizadas no cuidado à pessoa idosa com DM2, que articulem dimensões clínicas, subjetivas e organizacionais para favorecer melhores desfechos em saúde. 

Uma limitação do presente estudo refere-se à variabilidade de métodos e instrumentos utilizados na literatura para avaliar a qualidade de vida relacionada à saúde, o que dificulta a comparabilidade dos resultados, apesar do uso do SF-36, instrumento validado e amplamente empregado. Contudo, por se tratar de um instrumento genérico, sua menor sensibilidade a aspectos específicos do diabetes mellitus pode ter influenciado a ausência de correlações em alguns domínios.

A análise por caso disponível, sem imputação de dados, pode ter introduzido viés de completude, considerando a variação do número de participantes com exames laboratoriais disponíveis. Além disso, a variabilidade no número de casos válidos e a presença de dados ausentes inviabilizaram a realização de análises multivariadas, limitando o ajuste para potenciais fatores de confusão sociodemográficos. A ausência de medidas comportamentais, como adesão ao tratamento, dieta e atividade física, limita a interpretação dos achados, uma vez que esses fatores podem mediar a relação entre QVRS e controle metabólico. Observou-se ainda perda amostral em relação ao cálculo inicial, o que pode ter reduzido o poder estatístico, especialmente em análises correlacionais com dados ausentes.

Além disso, as associações identificadas apresentaram magnitude fraca, ainda que estatisticamente significativas, devendo ser interpretadas com cautela. O delineamento transversal também limita inferências causais. Apesar dessas restrições, o estudo possibilitou a compreensão de particularidades locais, conferindo relevância aos achados no contexto da APS.

Os achados deste estudo aprofundam a compreensão da relação entre QVRS e controle metabólico em pessoas idosas com DM2, contribuindo para a promoção de um envelhecimento mais saudável e fortalecendo a base de evidências, especialmente no campo da enfermagem, com potencial para orientar práticas assistenciais mais qualificadas na APS. Destaca-se a importância de abordagens centradas na pessoa, da construção de vínculos interpessoais, essenciais para a adesão ao tratamento e melhores desfechos clínicos^39^, e da valorização da saúde mental e da percepção subjetiva da doença na gestão do diabetes, indicando a necessidade de intervenções terapêuticas integradas que considerem fatores psicológicos e emocionais além dos parâmetros clínicos.

Os resultados refletem a realidade de pessoas idosas com DM2 atendidas em unidades de APS de um município do interior paulista e podem não ser diretamente generalizáveis para contextos com diferentes perfis sociodemográficos, organizacionais e de acesso à saúde. Contudo, a semelhança dos achados com estudos nacionais e internacionais sugere que as relações observadas entre QVRS e controle metabólico podem ocorrer em outras populações de pessoas idosas com DM2.

## Conclusão

Os resultados do estudo evidenciaram que as pessoas idosas com DM2 apresentaram uma QVRS satisfatória, embora coexistente com controle metabólico insatisfatório para importantes parâmetros clínicos. Observaram-se associações discretas, porém estatisticamente significativas, entre domínios específicos da QVRS, especialmente estado geral de saúde e saúde mental, e marcadores metabólicos. Embora de magnitude fraca, essas associações indicam que percepções subjetivas mais positivas de saúde e bem-estar mental se relacionaram a melhores níveis de controle glicêmico e perfil lipídico.

Esses achados reforçam a relevância de incorporar, na prática assistencial da APS, a avaliação sistemática da QVRS e dos aspectos psicossociais no acompanhamento de pessoas idosas com diabetes. A identificação de percepções negativas de saúde e de comprometimento da saúde mental pode funcionar como sinalizador clínico para maior risco de descontrole metabólico, subsidiando intervenções mais precoces e individualizadas.

No âmbito profissional, destaca-se o papel estratégico da enfermagem e das equipes multiprofissionais na condução de cuidados integrados, que articulem monitoramento clínico, ações educativas, apoio ao autocuidado e atenção à saúde mental. Tais estratégias podem favorecer maior adesão terapêutica e potencializar os resultados do manejo do diabetes nessa população.

Do ponto de vista das políticas públicas, os resultados apontam para a necessidade de fortalecimento de modelos de atenção que valorizem abordagens centradas na pessoa, com integração entre cuidado clínico e suporte psicossocial, especialmente no contexto do envelhecimento populacional e da elevada prevalência de doenças crônicas. Investimentos em qualificação das equipes da APS e em estratégias interdisciplinares podem contribuir para a melhoria dos desfechos em saúde e para a promoção de um envelhecimento mais saudável entre pessoas idosas com DM2.

## Data Availability

Todos os dados gerados ou analisados durante este estudo estão incluídos neste artigo publicado.

# Translation: Calidad de vida relacionada con la salud y control metabólico de personas mayores con diabetes mellitus^*^

**DOI:** 10.1590/1518-8345.8238.4935

**Authors:** Ana Laura Galhardo Figueira, Carla Auxiliadora Margarido, Maria Angélica Andreotti Diniz, Marília Pilotto de Oliveira, Lucia Bacalhau, Luciana Kusumota

**Affiliations:** 1 Universidade de São Paulo, Escola de Enfermagem de Ribeirão Preto, Centro Colaborador da OPAS/OMS para o Desenvolvimento da Pesquisa em Enfermagem, Ribeirão Preto, SP, Brasil.; 2 Prefeitura Municipal de Lorena, Secretaria de Saúde, Lorena, SP, Brasil.; 3 Prefeitura Municipal de Sertãozinho, Serviço de Atendimento Móvel de Urgência, Sertãozinho, SP, Brasil.; 4 Universidade Católica Portuguesa, Faculdade de Ciências da Saúde e Enfermagem, Lisboa, Portugal.

**Keywords:** Calidad de Vida, Diabetes Mellitus, Anciano, Atención Primaria de Salud, Salud Pública, Atención de Enfermería

## Abstract

**Objetivo:**

analizar la calidad de vida relacionada con la salud y su correlación con el control metabólico en personas mayores con diabetes mellitus tratadas en la Atención Primaria de Salud.

**Método:**

estudio observacional, transversal y correlacional con 264 personas mayores con diabetes mellitus tipo 2, atendidas en la Atención Primaria de Salud. La calidad de vida relacionada con la salud se evaluó utilizando el cuestionario *Short Form Health Survey* versión 2, y el control metabólico se evaluó mediante pruebas de laboratorio (glucosa en ayunas, HbA1c y perfil lipídico). Se aplicaron estadísticas descriptivas y la correlación de Spearman (α=0,05).

**Resultados:**

los participantes tenían una edad media de 70,23 años, el 70,5% eran mujeres y el 86% <9 años de escolaridad. El ámbito más comprometido de la calidad de vida relacionada con la salud fue el dolor, y el mejor rendimiento fue en los aspectos emocionales. Se observó una correlación inversa entre el estado general de salud y la HbA1c, así como entre la salud mental y el colesterol total, triglicéridos y LDL.

**Conclusión:**

las puntuaciones más altas de calidad de vida relacionada con la salud, especialmente en los ámbitos general de la salud y la salud mental, mostraron una correlación con mejores parámetros de control metabólico, lo que indica la relevancia de enfoques integrados en la atención de personas mayores con diabetes mellitus en la Atención Primaria de Salud.

## Destacados

**(1)** Envejecimiento y aumento de casos de diabetes mellitus tipo 2.

**(2)** Alta prevalencia de control metabólico inadecuado.

**(3)** La calidad de vida se relaciona con el control metabólico.

**(4)** Implicaciones para la práctica en Atención Primaria de Salud.

**(5)** La enfermería es esencial para la promoción, prevención y atención integral en la Atención Primaria de Salud.

## Introducción

Se estima que la población mundial mayor de 60 años, desde 1.000 millones en 2020, aumentará a 2.100 millones para 2050, con el 80% de ellos viviendo en países en desarrollo^1^. Este aumento presenta desafíos significativos para la sociedad y el sistema sanitario, especialmente en Brasil, donde el envejecimiento es uno de los más rápidos del mundo^2^.

Las personas mayores son más vulnerables al desarrollo de Enfermedades Crónicas No Transmisibles (DCNT)^3^, como la Diabetes Mellitus (DM), cuya prevalencia aumenta con la edad. En su publicación más reciente, la *International Diabetes Federation* estima que 589 millones de adultos entre 20 y 79 años viven con diabetes, lo que equivale a 1 de cada 9 personas en este grupo de edad. Se proyecta que esta cifra alcance los 853 millones para 2050^4^.

La DM es un grupo de trastornos metabólicos caracterizados por la infrautilización y una producción excesiva de glucosa, que conduce a la hiperglucemia^5^. Su tratamiento se basa en una dieta saludable, actividad física regular, mantenimiento de un peso corporal adecuado y abstinencia de fumar. Además, se indica el uso de medicamentos orales y, en muchos casos, de insulina para controlar los niveles de glucosa en sangre^6^. 

Cuando no se controla adecuadamente, la DM puede causar complicaciones que afectan negativamente a la calidad de vida (CV), manifestadas por debilidad física, reducción de la capacidad funcional, dolor, disminución de la vitalidad, dificultades sociales e inestabilidad emocional^7^. En este contexto, la calidad de vida, entendida como un constructo amplio y multidimensional, se analiza en el campo de la salud basándose en la Calidad de Vida Relacionada con la Salud (CVRS), que se centra en la percepción subjetiva del individuo sobre los efectos de la condición y el cuidado de salud en su funcionamiento físico, emocional y social^8^. Por ello, el presente estudio adopta la CVRS como un marco teórico para comprender los impactos de la DM y el control metabólico en la vida diaria de las personas mayores. 

El cuidado de las personas mayores con diabetes requiere un enfoque integral, que tenga en cuenta los aspectos médicos, psicológicos, funcionales y sociales, fundamentales para el establecimiento de objetivos terapéuticos realistas e individualizados, ya que estos factores pueden interferir en la autogestión de la enfermedad e influir en la CVRS^9^. Desde esta perspectiva, la Sociedad Brasileña de Diabetes establece objetivos clínicos y metabólicos específicos para esta población, con el objetivo de reducir complicaciones, morbilidad y mortalidad^10^ ^-^ ^11^. Sin embargo, un estudio brasileño en Atención Primaria de Salud (APS) indica que una parte significativa de las personas mayores con diabetes mellitus tipo 2 (DM2) no alcanzan los objetivos glucémicos recomendados, lo que evidencia que el control metabólico sigue siendo un desafío a este nivel de atención^12^.

Dada la complejidad de la enfermedad, la atención a las personas con DM requiere un seguimiento multiprofesional y continuo, con una coordinación eficaz de la atención. En este contexto, la PHC destaca como el nivel más adecuado para el seguimiento longitudinal, siendo la Estrategia de Salud Familiar (ESF) la principal política de organización PHC en Brasil, responsable de las acciones de promoción, prevención, tratamiento y rehabilitación^13^ ^-^ ^14^. Insertado en esta estructura, el equipo de enfermería desempeña un papel central en la continuidad de la atención, especialmente en el desarrollo de acciones educativas, el seguimiento clínico y el fortalecimiento del autocuidado, contribuyendo decisivamente a la gestión integral de la DM^15^.

A pesar del reconocimiento de la relevancia de la CVRS, todavía existen pocos estudios que investiguen, de manera integrada, la relación entre los diferentes dominios de la CVRS y múltiples parámetros de control metabólico en personas mayores con DM. Esto limita la incorporación sistemática de esta dimensión subjetiva en la planificación y calificación del cuidado.

Así, considerando la relevancia de la CVRS como dimensión complementaria a los indicadores clínicos y la falta de evidencia en el contexto de la APS, el objetivo de este estudio fue analizar la CVRS y su correlación con el control metabólico de personas mayores con DM2 en la APS en un municipio del interior de São Paulo, Brasil. 

## Método

### Diseño del estudio

Se trata de un estudio observacional, analítico y correlacional con un diseño transversal. Se basa en las directrices del *Strengthening the Reporting of Observational Studies in Epidemiology* ****(STROBE).

### Lugar y periodo de la recogida de datos

El estudio se llevó a cabo en 11 unidades de ESF de un municipio del interior del estado de São Paulo, Brasil, entre junio y octubre de 2022.

### Población

La población estaba compuesta por 1.113 personas mayores que informaron tener DM en el registro individual del sistema nacional de registro de salud, vinculado a una de las unidades de ESF del municipio en cuestión. Este número representa el total encontrado en el sistema electrónico en junio de 2021, el año en que se realizó la encuesta diagnóstica de los datos para planificar la encuesta.

### Criterios de selección

Personas con diagnóstico médico de DM2, de al menos 60 años, sometidas a tratamiento farmacológico con Medicamentos Antidiabéticos Orales (ADO) y/o insulina, funciones cognitivas preservadas (evaluadas mediante el Mini-Examen del Estado Mental - MMSE)^16^ ^)^ y con habilidades de comunicación verbal. La puntuación final del MMSE puede oscilar entre 0 y 30 puntos^17^ y se utilizaron puntuaciones de corte basadas en la escolarización, considerando los siguientes puntos: 18 (analfabeto), 21 (1-3 años de escolarización), 24 (4-7 años de escolarización) y 26 (> 7 años de escolarización)^18^.

### Definición de la muestra

La identificación de posibles participantes se realizó mediante el sistema nacional de historiales médicos, utilizando los filtros: edad igual o superior a 60 años y DM. Se decidió incluir a todos los individuos elegibles posibles en el periodo de recogida, para maximizar el poder estadístico. Considerando una población de 1.113 personas mayores que reportaron tener DM (número total antes de que se confirmara el diagnóstico de DM2), se estableció un nivel de confianza del 95% y un error máximo tolerado del 5%, lo que resultó en un tamaño mínimo de muestra estimado de aproximadamente 285 individuos. La muestra final constó de 264 participantes, y no fue posible aumentar este número debido al periodo previamente definido en el calendario de investigación, así como a limitaciones operativas y logísticas. Aunque ligeramente inferior al número estimado, este tamaño de muestra mantiene una representatividad suficiente para la población objetivo, permitiendo realizar análisis con la adecuada robustez estadística. Además, tras revisar los registros de los participantes que aceptaron participar en la investigación, se descubrió que no todos tenían un diagnóstico médico confirmado de DM2, lo que contribuyó a las pérdidas y redujo el número real de individuos elegibles entre los 1.113 considerados inicialmente.

### Variables del estudio

El estudio incluyó las siguientes variables: 1. Variables sociodemográficas: género, edad, color de piel, estado civil, educación, ocupación actual, número de residentes en el hogar e ingresos mensuales de la familia. 2. Calidad de vida relacionada con la salud (CVRS): ocho dominios del *Short Form 36 Health Survey* versión 2 (SF-36v2^®^). 3. Control metabólico: glucosa plasmática en ayunas, hemoglobina glicada A1c (HbA1c), colesterol total, colesterol de alta densidad (HDL), colesterol de baja densidad (LDL) y triglicéridos.

### Instrumentos utilizados para la recopilación de información

Los datos sociodemográficos se recopilaron utilizando un instrumento desarrollado para este propósito. La CVRS se evaluó utilizando el SF-36v2^®^, un cuestionario fiable y sensible a los cambios en la salud, que evalúa múltiples ámbitos de la calidad de vida. El cuestionario se aplica rápidamente, se valida para la población brasileña y tiene propiedades psicométricas adecuadas, como la consistencia interna y la validez del constructo, que también se han demostrado en estudios con personas mayores^19^.

El SF-36 consta de 35 ítems y una pregunta que compara la salud actual con la de hace un año. Evalúa la calidad de vida percibida en las últimas cuatro semanas, abarcando aspectos físicos y mentales, tanto negativos (limitaciones) como positivos (bienestar). Los ítems se distribuyen en ocho dominios: capacidad funcional (CF - 10 ítems), aspectos físicos (AF - 4), dolor (2), estado general de salud (EGS - 5), vitalidad (VT - 4), aspectos sociales (AS - 2), aspectos emocionales (AE - 3) y salud mental (SM - 5). Estos dominios están organizados en dos componentes: físico (CF, AF, dolor y EGS) y mental (VT, AS, AE y SM). En el presente estudio, se presentaron las puntuaciones medias de cada dominio, que iban desde 0 (peor CVRS) hasta 100 (mejor CVRS)^20^. El número de licencia QM057367 se obtuvo para el uso de SF-36v2^®^ del QualityMetric Inc.

Las variables sociodemográficas y de la CVRS fueron autoinformadas por los participantes. Los resultados de laboratorio para la evaluación del control metabólico se obtuvieron a partir de historiales médicos electrónicos, considerando pruebas realizadas hasta tres meses antes o después de la recogida de datos. No hubo estratificación en la fecha exacta de las pruebas en este intervalo, lo que puede introducir heterogeneidad debido a posibles cambios terapéuticos, un aspecto considerado en la interpretación de los resultados. Las pruebas se realizaron en un único laboratorio de referencia de la red pública municipal, contribuyendo a la estandarización de los métodos analíticos y a la reducción de la variabilidad. La recogida de muestras siguió la rutina del servicio, realizada por profesionales formados, según protocolos institucionales, asegurando una mayor uniformidad de los datos.

Para evaluar estas últimas variables, se siguieron los estándares establecidos por consenso y/o directrices, y los siguientes valores se clasificaron como “normal” o “alterado”: glucosa plasmática en ayunas (normal ≤ 130 mg/dl y alterada > 130 mg/dl); HbA1c (normal < 7,5% y alterada ≥ 7,5%)^11^; colesterol total (normal < 200 mg/dl y alterado ≥ 200 mg/dl)^21^; triglicéridos (normales < 150 mg/dl y alterados ≥ 150 mg/dl)^22^; HDL (normal > 45 mg/dl y alterado ≤ 45 mg/dl); LDL (normal < 100 mg/dl y alterado ≥ 100 mg/dl)^21^.

### Recogida de datos

La recogida de datos fue realizada por un solo investigador entrenado para reducir la variabilidad entre observadores y el sesgo de información. El uso de un instrumento validado (SF-36v2^®^) y datos de laboratorio registrados en el sistema electrónico buscaba aumentar la fiabilidad de las mediciones. Las entrevistas se realizaron en el domicilio del participante o, cuando fue posible, en la unidad de salud, según disponibilidad y preferencia, en un entorno reservado y sin la presencia de terceros, con el fin de minimizar la interferencia externa y garantizar la privacidad y el confort, y el SF-36 es un instrumento estructurado de autoinforme que contribuye a la estandarización de las respuestas independientemente del lugar de aplicación.

Los instrumentos de estudio, impresos en papel, tenían las respuestas rellenadas con tinta y eran aplicados por el investigador responsable. Se pidió a quienes aceptaron participar en el estudio que firmaran el formulario de consentimiento informado (FCI), y se guardó una copia con el participante.

### Procesamiento y análisis de datos

Los datos se introducían en hojas de cálculo de Microsoft Excel^®^ con doble entrada para garantizar la coherencia. El análisis se realizó utilizando el *software* R (versión 4.3.1), utilizando estadísticas descriptivas para caracterizar variables sociodemográficas, parámetros de control metabólico y dominios de la CVRS, presentados en frecuencias absolutas y relativas, medias (con desviación estándar) y medianas (con valores mínimos y máximos). El análisis bivariado se realizó utilizando el Coeficiente de Correlación de Spearman, con un nivel de significación del 5% (α = 0,05). Las variables con datos faltantes se mantuvieron en el análisis descriptivo indicando el número válido (n). No se realizó imputación de datos, optando por el análisis por caso disponible para evitar la introducción de sesgo.

No se utilizaron modelos multivariantes debido a la variabilidad en el número de casos válidos entre las diferentes pruebas de laboratorio, la ausencia de datos y la naturaleza transversal del estudio, factores que podían comprometer la estabilidad y robustez de los modelos. Se reconoce que las características sociodemográficas pueden actuar como posibles factores de confusión, un aspecto considerado en la interpretación de los resultados y discutido como una limitación del estudio.

### Consideraciones éticas 

El estudio se llevó a cabo conforme a las directrices éticas nacionales e internacionales y fue aprobado por el Comité de Ética en Investigación de la Escuela de Enfermería de Ribeirão Preto de la Universidade de São Paulo, obteniendo la aprobación bajo el Dictamen N.º 5.349.953.

## Resultados

Durante el periodo de recogida de datos, se incluyeron 1.113 participantes elegibles para la investigación. El flujo de cribado e inclusión se muestra en la Figura 1.

**Figura 1:** Proceso de elegibilidad y selección de los participantes del estudio

*n = Número de encuestados; ^†^DM2 = Diabetes Mellitus tipo 2

La muestra estaba compuesta predominantemente por mujeres, con una edad media de 70,23 años (DS=6,84), la mayoría de las cuales pertenecían al grupo de edad de 60 a 69 años. Había una prevalencia de personas blancas autodeclaradas, con baja educación y retiradas o sin actividad remunerada. La mayoría vivía con otros residentes, con una media de hasta tres personas por hogar. La renta familiar se concentraba mayormente en el rango de uno a tres salarios mínimos. La Tabla 1 presenta la caracterización sociodemográfica de los participantes.

En la evaluación de la CVRS, hubo una variación en las medias en los dominios de personas mayores con DM2, con mayor deterioro del dominio del dolor y mejor CVRS en la evaluación del dominio AE (Tabla 2).

**Tabla 1:** Caracterización sociodemográfica de los participantes del estudio (n = 264). Lorena, SP, Brasil 2022

Variables sociodemográficas	Media (DE✱)	n† (%‡)	Mediana (mínimo-máximo)

**Género**			
Masculino		78 (29,5)	
Femenino		186 (70,5)	

**Edad (años completos)**	70,23 (6,84)		70,00 (60,00 – 94,00)

**Grupo de edad (en años)**			
60-69		130 (49,2)	
70-79		105 (39,8)	
80-89		25 (9,5)	
90-99		4 (1,5)	

**Color de la piel**			
Blanco		171 (64,8)	
Negro		36 (13,6)	
Amarillo		1 (0,4)	
Marrón		56 (21,2)	

**Estado civil**			
Soltera(o)		28 (10,6)	
Casada(o)/En pareja (unión libre)		126 (47,7)	
Separada(o)/Divorciada(o)		31 (11,8)	
Viuda(o)		79 (29,9)	

**Educación (años completos de estudio)**	4,57 (3,52)		4,00 (0 – 19,00)

**Nivel de educación (en años)**			
Bajo (< 9)		227 (86,0)	
Medio (9-12)		28 (10,6)	
Alto (>12)		9 (3,4)	

**Ocupación actual**			
Retirada(o) que mantienen una relación laboral		5 (1,9)	
Retirada(o)/pensionista/trabaljo en casa (sin remuneración)		251 (95,1)	
Desempleada(o)		8 (3,0)	

**Vive solo**			
Sí		53 (20,1)	
No		211 (79,9)	

**Número total de personas que viven en la casa**			
1-3 personas	2,55 (1,39)	214 (81,1)	2,00 (1,00-8,00)
4-6 personas		45 (17,0)	
7-9 personas		5 (1,9)	

**Ingreso mensual familiar (n†=262) (en reales)§**			
< 1 salario	2.139,46 (1.566,81)	16 (6,1)	
1-3 salarios		234 (89,3)	
4 salarios o más		12 (4,6)	


*DE = Desviación estándar; ^†^n = Número de encuestados; ^‡^% = Porcentaje, ^§^Salario mínimo vigente = R$ 1.212,00, Brasil, 2022

**Tabla 2:** Resultados descriptivos de los ocho dominios de la Calidad de Vida Relacionada con la Salud de los participantes del estudio (n = 264). Lorena, SP, Brasil 2022

Dominio	Media (DE*)	Mediana (mínimo-máximo)
Capacidad Funcional	65,41 (28,37)	75,00 (0-100)
Aspectos Físicos	84,42 (30,84)	100,00 (0-100)
Dolor	59,94 (31,20)	61,00 (0-100)
Estado General de Salud	62,97 (24,06)	67,00 (0-100)
Vitalidad	64,54 (30,52)	75,00 (0-100)
Aspectos Sociales	82,95 (25,74)	100,00 (0-100)
Aspectos Emocionales	88,57 (25,62)	100,00 (0-100)
Salud Mental	71,50 (28,46)	80,00 (0-100)

^*^ DE = Desviación estándar

El análisis de los resultados del control metabólico reveló cambios en la media de la mayoría de las pruebas evaluadas. Se observó que la mayoría de los participantes presentaban niveles alterados de glucosa en ayunas y LDL, lo que indica un deterioro en el control de estas variables. También es destacable la marcada dispersión de los valores de glucosa en ayunas, evidenciada por la amplia variación entre los valores mínimo y máximo (60-416 mg/dl), lo que refleja la heterogeneidad en el control glucémico entre las personas mayores evaluadas (Tabla 3).

**Tabla 3:** Caracterización de los resultados del control metabólico de los participantes en el estudio (n = 264). Lorena, SP, Brasil 2022

Variables de control metabólico	Media (DE*)	n^†^ (%^‡^)	Mediana (mínimo-máximo)

**Glucosa en Ayunas (mg/dl^§^) (n^†^=226)**			
Normal (<130)	151,71 (67,77)	108 (47,8)	132,00 (60,00-416,00)
Alterada (≥130)		118 (52,2)	

**Hemoglobina Glicosilada (%^‡^) (n^†^=230)**			
Normal (<7,5)	7,96 (2,30)	124 (53,9)	7,20 (4,50-16,90)
Alterada (≥7,5)		106 (46,1)	

**Colesterol Total (mg/dl^§^) (n^†^=222)**			
Normal (<200)	199,11 (49,65)	115 (51,8)	196,50 (61,00-396,00)
Alterado (≥200)		107 (48,2)	

**Triglicéridos (mg/dl^§^) (n^†^=221)**			
Normal (<150)	169,20 (95,80)	114 (51,6)	147,00 (46,00-730,00)
Alterado (≥150)		107 (48,4)	

**HDL (mg/d^§|^) (n^†^=211)**			
Normal (>45)	50,15 (14,75)	125 (59,2)	49,00 (22,00-137,00)
Alterado (≤45)		86 (40,8)	

**LDL (mg/dl^§^) (n^†^=212)**			
Normal (<100) Alterado (≥100)	115,50 (43,12)	80 (37,7)	147,00 (46,00-305,00)
		132 (62,3)	


*DE = Desviación estándar; ^†^n = Número de encuestados; ^‡^% = Porcentaje; ^§^mg/dl = Miligramos/decilitro

El análisis estadístico reveló correlaciones inversas de magnitud débil, aunque estadísticamente significativas, entre dominios específicos de la CVRS y marcadores metabólicos. Las personas mayores con DM2 con mejores percepciones de su EGS y SM obtuvieron mejores resultados en pruebas de laboratorio, lo que sugiere que niveles más altos de CVRS se correlacionan con un control metabólico más adecuado (Tabla 4).

**Tabla 4:** Correlación entre las variables Calidad de Vida Relacionada con la Salud y Control Metabólico de los participantes en el estudio (n = 264). Lorena, SP, Brasil 2022

Variables de control metabólico	Capacidad Funcional	Aspectos Físicos	Dolor	Estado General de Salud	Vitalidad	Aspectos Sociales	Aspectos Emocionales	Salud Mental
r^*^ (*p* ^*†*^ , - valor)								
Glucosa en Ayunas (n^‡^=226)	0,0891 (0,1819)	-0,0231 (0,6962)	-0,0178 (0,7907)	-0,1259 (0,0588)	-0,1006 (0,1317)	0,0008 (0,9903)	-0,0029 (0,9656)	-0,1266 (0,0573)
Hemoglobina Glicosilada (n^‡^=230)	-0,0486 (0,4630)	-0,0994 (0,1328)	-0,1067 (0,1066)	-0,1525 (0,0207^†^)	-0,1171 (0,0763)	-0,0861 (0,1932)	-0,0861 (0,1932)	-0,1044 (0,1145)
Colesterol total (n^‡^=222)	-0,0787 (0,2432)	-0,0629 (0,3509)	-0,0988 (0,1423)	-0,0545 (0,4190)	-0,0925 (0,1697)	-0,0976 (0,1474)	-0,0482 (0,4747)	-0,2635 (0,0100^†^)
Triglicéridos (n^‡^=221)	-0,0346 (0,6091)	0,0258 (0,7026)	0,0369 (0,5856)	-0,0290 (0,6681)	-0,0229 (0,6681)	0,0323 (0,6333)	-0,1070 (0,1128)	-0,1711 (0,0108^†^)
HDL (n^‡^=211)	-0,0986 (0,1536)	-0,0986 (0,1536)	-0,0616 (0,3737)	-0,0139 (0,8408)	0,0044 (0,9489)	-0,0825 (0,2326)	-0,0196 (0,7776)	0,0013 (0,9849)
LDL (n^‡^=212)	-0,0639 (0,3544)	-0,0515 (0,4561)	-0,0927 (0,1786)	-0,0201 (0,7709)	-0,0882 (0,2010)	-0,0545 (0,4302)	0,0407 (0,5557)	-0,2128 (0,0100^†^)

^*^ r = Coeficiente de correlación de Spearman; ^†^p = Nivel de significación (los valores de p ≤ 0,05 se consideraron estadísticamente significativos y se indican con ^†^); ^‡^n = Número de encuestados

## Discusión

Este estudio dio lugar a tres hallazgos considerables: (1) Las personas mayores con DM2 presentaron una CVRS satisfactoria, aunque con un deterioro moderado en algunos dominios, especialmente en el dolor, mientras que el mejor rendimiento se observó en el dominio AE; (2) Control metabólico comprometido entre los participantes; (3) Los aspectos subjetivos de la percepción de la salud mostraron una asociación estadística con los parámetros de control metabólicos. Sin embargo, debido al diseño transversal, estos hallazgos no permiten establecer relaciones causales, indicando únicamente la existencia de asociaciones entre la CVRS y el control metabólico.

En cuanto al primer hallazgo, los dominios de la CVRS presentaron generalmente puntuaciones altas, destacando AE como el mejor desempeño, mientras que el dominio del dolor fue el más comprometido, lo que evidencia su impacto negativo en la CVRS de personas mayores con DM2 seguidas en la APS. Este resultado difiere de los estudios internacionales realizados con adultos con DM2, en los que las puntuaciones más altas se concentraron en el dominio SM y las más bajas en AF, lo que indica un mayor impacto funcional de la enfermedad. Estas divergencias pueden reflejar diferencias de edad, socioculturales, clínicas y de acceso a los servicios de salud, que influyen en la percepción del bienestar y las limitaciones diarias^23^, especialmente al comparar los sistemas de salud con la APS brasileña, guiados por un seguimiento regular y coordinación de la atención.

En cuanto al segundo hallazgo, los resultados del presente estudio corroboran la literatura nacional e internacional, indicando que el control metabólico en personas mayores con DM2 tiende a ser insatisfactorio. Un estudio multicéntrico realizado en China, con 2.736 personas mayores, mostró que solo el 23% alcanzó HbA1c < 7% y el 4,6% logró simultáneamente objetivos metabólicos^24^. En Brasil, los datos de Ribeirão Preto (SP) revelaron una media de HbA1c de 7,2 ± 1,7%, con un 74,1% de ancianos por debajo del 8%, lo que evidencia las dificultades en el manejo metabólico de esta población^12^. La convergencia de estos hallazgos sugiere que un control metabólico inadecuado de personas mayores con DM2 es un desafío global, pero que, en la APS brasileña, puede estar relacionado con la adopción de conductas clínicas individualizadas, compatibles con las necesidades y características de la persona mayor.

Respecto al tercer hallazgo, que se refiere a la asociación entre los aspectos físicos de la CVRS y los parámetros de control metabólicos, se observó que, aunque el envejecimiento y la diabetes mellitus están asociados con disminuciones en la función física y una mayor prevalencia de dolor crónico^25^ ^-^ ^27^, no hubo correlaciones significativas entre los dominios CF, AF y dolor y el control metabólico. Este hallazgo puede estar relacionado con el seguimiento regular en la APS, el uso de terapia adecuada, estrategias de autocuidado o la influencia de comorbilidades, lo que indica que las condiciones físicas desfavorables no siempre se reflejan en un peor control metabólico en este contexto.

También en relación con los dominios físicos de la CVRS, se destaca el EGS, cuya correlación inversa con la HbA1c indica que una percepción más negativa de la salud se asocia con niveles más altos de glucosa en sangre, reflejando una mayor dificultad para mantener el control metabólico. Este hallazgo se corrobora con un estudio realizado en India con personas con DM2, que, tras seis meses de educación en autogestión, mostró una mejora significativa en los niveles de HbA1c y en las puntuaciones del componente físico del SF-36, evidenciando una asociación entre el control glucémico y la percepción del bienestar, así como por un estudio que identificó una peor calidad de vida física entre personas mayores iraníes con diabetes mal controlada^28^.

En cuanto a los aspectos mentales de la CVRS, los dominios VT, AS y AE no presentaron correlación con el control metabólico de personas mayores con DM2 en este estudio, aunque la literatura señala una asociación entre un control glucémico inadecuado y peores puntuaciones de vitalidad, relacionada con una mayor fatiga^29^. La evidencia también indica que la participación social de las personas mayores con DM está limitada por factores como la movilidad y el autocuidado, y puede influir indirectamente en el control metabólico^30^, así como en los síntomas depresivos y el estrés diabético, que comprometen la adherencia al tratamiento^31^. Se observó una correlación inversa entre el dominio de la SM y los niveles de colesterol total, triglicéridos y LDL, lo que indica una asociación entre mejores puntuaciones de SM y un perfil lipídico más favorable, sin permitir inferencia causal, considerando el diseño transversal. Además, un estudio con una población diferente identificó que un perfil lipídico desfavorable se asociaba con un mayor riesgo futuro de depresión, ansiedad y trastornos relacionados con el estrés^32^.

La relación inversa entre las puntuaciones de EGS y HbA1c, así como entre el dominio SM y los niveles totales de colesterol, triglicéridos y LDL, sugiere que los aspectos subjetivos del bienestar están más asociados al control metabólico que a limitaciones físicas. En este contexto, la CVRS desempeña un papel central, ya que los cambios en la percepción de la salud y el bienestar emocional pueden influir en el autocuidado^33^, incluida la adherencia terapéutica, reflejándose en mejores indicadores metabólicos^34^ ^-^ ^36^. Corroborando estos hallazgos, un metaanálisis con adultos de mediana edad y personas mayores con DM2 demostró una reducción significativa de HbA1c tras intervenciones psicológicas, indicando que las estrategias orientadas a los aspectos psicológicos contribuyen al control glucémico, aunque aún se necesitan estudios para identificar los enfoques más efectivos^37^.

En el contexto de la APS, los resultados destacan la importancia de la enfermería como parte integral de un equipo multidisciplinar en el cuidado de personas mayores con DM2, especialmente en el seguimiento longitudinal, el seguimiento clínico y las acciones educativas orientadas al autocuidado^38^. La identificación de aspectos subjetivos de la CVRS asociados al control metabólico refuerza el papel estratégico de la enfermería en el fortalecimiento del vínculo y la cualificación de la atención ofrecida.

Sin embargo, el control metabólico en la APS no depende solo de las características individuales o subjetivas de las personas mayores, sino también de factores contextuales relacionados con la organización de los servicios, como la disponibilidad de pruebas de laboratorio, la regularidad del seguimiento clínico, la continuidad de la atención y la heterogeneidad entre unidades. Estos aspectos estructurales y organizativos pueden influir tanto en indicadores metabólicos como en la percepción de la salud y la calidad de vida, reforzando la necesidad de enfoques integrales y contextualizados en el cuidado de personas mayores con DM2, que articulen dimensiones clínicas, subjetivas y organizativas para favorecer mejores resultados en salud.

Una limitación del presente estudio se refiere a la variabilidad de los métodos e instrumentos utilizados en la literatura para evaluar la calidad de vida relacionada con la salud, lo que dificulta la comparación de los resultados, a pesar del uso del SF-36, un instrumento validado y ampliamente utilizado. Sin embargo, al ser un instrumento genérico, su menor sensibilidad a aspectos específicos de la diabetes mellitus puede haber influido en la ausencia de correlaciones en algunos dominios.

El análisis por caso disponible, sin imputación de datos, pudo haber introducido un sesgo de completitud, considerando la variación en el número de participantes con pruebas de laboratorio disponibles. Además, la variabilidad en el número de casos válidos y la ausencia de datos hicieron imposible realizar análisis multivariantes, limitando el ajuste por posibles factores sociodemográficos de confusión. La ausencia de medidas conductuales, como la adhesión al tratamiento, la dieta y la actividad física, limita la interpretación de los hallazgos, ya que estos factores pueden mediar la relación entre la CVRS y el control metabólico. También se observó una pérdida muestral en relación con el cálculo inicial, lo que pudo haber reducido el poder estadístico, especialmente en análisis correlacionales con datos faltantes.

Además, las asociaciones identificadas presentaban magnitud débil, aunque estadísticamente significativas, y deben interpretarse con cautela. El diseño transversal también limita las inferencias causales. A pesar de estas restricciones, el estudio permitió comprender particularidades locales, dando relevancia a los hallazgos en el contexto de la APS.

Los hallazgos de este estudio profundizan en la comprensión de la relación entre la CVRS el control metabólico en personas mayores con DM2, contribuyendo a promover un envejecimiento más saludable y reforzando la base de evidencia, especialmente en el ámbito de la enfermería, con el potencial de guiar prácticas de atención más cualificadas en la APS. Se destaca la importancia de enfoques centrados en la persona, la construcción de vínculos interpersonales, que son esenciales para la adherencia al tratamiento y mejores resultados clínicos^39^, y la valoración de la salud mental y la percepción subjetiva de la enfermedad en el manejo de la diabetes, lo que indica la necesidad de intervenciones terapéuticas integradas que tengan en cuenta factores psicológicos y emocionales además de los parámetros clínicos.

Los resultados reflejan la realidad de las personas mayores con DM2 atendidas en unidades de APS en un municipio del interior de São Paulo y pueden no ser directamente generalizables a contextos con diferentes perfiles sociodemográficos, organizativos y de acceso a perfiles de salud. Sin embargo, la similitud de los hallazgos con estudios nacionales e internacionales sugiere que las relaciones observadas entre la CVRS y el control metabólico pueden darse en otras poblaciones de personas mayores con DM2.

## Conclusión

Los resultados del estudio mostraron que las personas mayores con DM2 tenían una CVRS satisfactoria, aunque coexistían con un control metabólico insatisfactorio de parámetros clínicos importantes. Se observaron asociaciones discretas, pero estadísticamente significativas entre dominios específicos de la CVRS, especialmente la salud general y la salud mental, y los marcadores metabólicos. Aunque de magnitud débil, estas asociaciones indican que percepciones subjetivas más positivas sobre la salud y el bienestar mental se relacionaban con mejores niveles de control glucémico y perfil lipídico.

Estos hallazgos refuerzan la relevancia de incorporar la evaluación sistemática de la CVRS y los aspectos psicosociales en el seguimiento de personas mayores con diabetes en la práctica de cuidados de la APS. La identificación de percepciones negativas sobre la salud y la salud mental comprometida puede actuar como una señal clínica de un mayor riesgo de descontrol metabólico, apoyando intervenciones más tempranas e individualizadas.

En el ámbito profesional, se destaca el papel estratégico de los equipos de enfermería y multiprofesionales en la realización de la atención integrada, que articula la monitorización clínica, las acciones educativas, el apoyo al autocuidado y la atención de la salud mental. Estas estrategias pueden favorecer una mayor adherencia terapéutica y mejorar los resultados del manejo de la diabetes en esta población.

Desde el punto de vista de las políticas públicas, los resultados apuntan a la necesidad de fortalecer los modelos de atención que valoren enfoques centrados en la persona, con integración entre la atención clínica y el apoyo psicosocial, especialmente en el contexto del envejecimiento poblacional y la alta prevalencia de enfermedades crónicas. Las inversiones en la cualificación de equipos de la APS y en estrategias interdisciplinarias pueden contribuir a la mejora de los resultados de salud y a la promoción de un envejecimiento más saludable entre las personas mayores con DM2.

## Data Availability

Todos los datos generados o analizados durante este estudio están incluidos en este artículo publicado.
